# Histone lactylation maintains bovine early embryo development via regulating embryonic genome activation

**DOI:** 10.1186/s40104-026-01398-8

**Published:** 2026-04-23

**Authors:** Wenjie Yu, Yi Xia, Wenzhuo Wang, Zhiyuan Yang, Zhengqiu Liu, Xinqi Gan, Mengya Zhang, Beibei Zhou, Yunsheng Li, Yunhai Zhang, Zubing Cao

**Affiliations:** Anhui Province Key Laboratory of Local Livestock and Poultry, Genetical Resource Conservation and Germplasm Innovation, College of Animal Science and Technology, Anhui Agricultural University, No.130 West Changjiang Road, Hefei, 230036 China

**Keywords:** Cattle, Embryonic genome activation, Histone lactylation, In vitro fertilization

## Abstract

**Background:**

Early embryo loss is an important factor affecting the reproductive capacity of cattle. Recent studies have revealed that during the process of embryonic genome activation (EGA), epigenetic modification, such as histone lactylation remodeling, is crucial for early embryonic development. However, the effects of histone lactylation on early embryonic development in bovines and the related mechanisms remain unknown. In this study, in vitro fertilized embryos were utilized to investigate the effects of histone lactylation on EGA and early embryo development in cattle.

**Results:**

Histone lactylation, including pan-lysine lactylation, histone H3 lysine 9 lactylation, and histone H3 lysine 18 lactylation, occurred mainly in the nucleus and significantly decreased from the 8-cell stage to the morula stage and increased from the morula stage to the blastocyst stage. Decreased or increased levels of histone lactylation induced by GSKA or sodium lactate supplementation inhibited early bovine embryo development and blastocyst lineage differentiation. Furthermore, single-cell RNA sequencing data and 5-ethynyluridine staining revealed that a reduction in histone lactylation levels altered the expression of genes associated with DNA transcription and RNA polymerase activity, thereby impairing EGA. Importantly, β-nicotinamide mononucleotide rescued the inhibitory effects of GSKA supplementation on bovine EGA and early embryonic development.

**Conclusions:**

Histone lactylation maintains early bovine embryo development by regulating EGA. Our findings provide a theoretical reference for addressing early embryo loss and thereby increasing the reproductive capacity of cattle.

**Supplementary Information:**

The online version contains supplementary material available at 10.1186/s40104-026-01398-8.

## Background

Cattle, as important economic animals, are closely related to livestock production and human life. Beef, milk, and related products play significant roles in improving the dietary structure and health of people. However, low fertility is an important factor affecting the production efficiency and economic benefits of the cattle industry. Studies have shown that early embryo death is the main cause of low fertility [1], with approximately 37% of embryos dying within one week after fertilization [2]. Abnormal embryo development, even death leading to embryo loss during early pregnancy, is a key factor restricting the reproductive capacity and breeding process of cattle.

After the follicle ruptures and ovulation occurs, the mature oocyte combines with the sperm to form a fertilized egg. Approximately 16 h after fertilization, the male and female pronuclei begin to closely fuse, indicating that the normal fertilization process of the embryo is complete, leading to subsequent development [3, 4]. At the early stage of fertilization, embryo development is dependent mainly on maternal mRNA and proteins. As development progresses, the embryonic genome gradually becomes activated and begins to initiate the transcription of its own genes [5]. Studies have shown that the timing of embryonic genome activation (EGA) differs among different species, for example, at the 2-cell embryo stage for mice [6], 8/16-cell embryo stage for cattle [5], 8/16-cell embryo stage for sheep [7], and 4-cell embryo stage for pigs [8]. EGA involves a series of complex events, such as the degradation of maternal factors, the expression of embryo-specific transcription factors, and epigenetic remodeling [9]. Crosstalk between metabolic processes and epigenetic regulation, particularly between lactate and histone lactylation, is essential for EGA and early development [10, 11].

Lactic acid is an indispensable metabolic substrate for early embryonic development. The addition of lactic acid to embryo in vitro culture (IVC) medium can increase the blastocyst rate of somatic cell nuclear transfer embryos by improving early embryo development [12]. Lactic acid is also a key signaling molecule that serves as a substrate for histone lactylation. Lactic acid deficiency leads to the significant suppression of histone H3 lysine 18 lactylation (H3K18la) and the failure of zygotic genome activation, resulting in the arrest of mouse embryo development at the 2-cell stage [13]. Moreover, lactic acid metabolism is disrupted and a significant decrease in the blastocyst rate is caused by reduced pan-lysine lactylation (pan Kla), histone H3 lysine 9 lactylation (H3K9la), and H3K18la modification levels under hypoxic conditions [14]. A previous study revealed that pan Kla occurs within the nucleus of 8-cell embryos during the critical period of EGA and that decreased levels of lactic acid may inhibit the developmental efficiency of bovine embryos [11]. Therefore, studying the function and mechanism of histone lactylation during early bovine embryonic development is highly important.

In this study, we investigated the role and potential mechanism of histone lactylation in early bovine embryonic development. We found that histone lactylation changed dynamically and maintained the process of early embryo development by regulating EGA. This study provides a theoretical basis for addressing the abnormal development of early bovine embryos and represents a theoretical reference for optimizing the bovine reproduction industry.

## Methods

### Chemicals

Unless otherwise stated, all the reagents used were purchased from Sigma/MERCK (Sigma-Aldrich, St. Louis, MO, USA).

### In vitromaturation (IVM) of bovine oocytes

Bovine ovaries were collected from a slaughterhouse, and follicular fluid was collected from 3–8 mm antral follicles. Oocytes that displayed a homogeneous cytoplasm and were packed with a minimum of three layers of cumulus cells were selected with a stereomicroscope and cultured in 400 μL of IVM medium (Tissue Culture Medium 199‌ supplemented with 10% fetal bovine serum (Gibco, Grand Island, NY, USA), 1% penicillin‒streptomycin sulfate, epidermal growth factor (30 ng/mL), follicle-stimulating hormone (10 IU/mL; Ningbo Second Hormone Factory, Ningbo, China), luteinizing hormone (10 IU/mL; Ningbo Second Hormone Factory), and sodium pyruvate (100 mmol/L; Gibco)). Then, the oocytes were cultured in an incubator (CIB-191TX; Suzhou Jiemei Electronic Co., Ltd., Suzhou, China) at 38.5 °C with 5% CO₂ and 5% O₂ for 22–24 h.

### In vitrofertilization (IVF) and embryo IVC

Mature oocytes were subsequently washed three times with prewarmed SOF-FERT solution [15] and then placed in a fertilization dish with 300 μL of IVF medium (IVF Bioscience, Falmouth, UK). A sperm suspension was added to fertilization dishes containing oocytes, followed by incubation at 38.5 °C with 5% CO₂ for 9 to 12 h. The final concentration of sperm was 5 × 10^6^/mL. After fertilization, the cumulus cells were removed by aspiration with a pipette, and potential fertilized eggs containing polar bodies were subsequently selected and transferred to 400 μL of IVC medium (IVF Bioscience) in an incubator at 38.5 °C with 5% CO₂ and 5% O₂.

### Alteration of histone lactylation by sodium lactate (NaLa), GSKA, and β-nicotinamide mononucleotide (NMN)

NaLa was used to specifically increase the level of histone lactylation [16]. A 60% (w/w) NaLa syrup was stored at 4 °C in the dark. Embryos were treated with 0, 5, or 10 mmol/L NaLa. GSKA (MedChemExpress, Shanghai, China) was used to specifically decrease the level of histone lactylation [14]. GSKA was diluted with DMSO to prepare a 200 nmol/L stock solution. Embryos were treated with 0, 100, 150, or 200 pmol/L GSKA. NMN was used to specifically increase the level of histone lactylation [17]. NMN was diluted with DMSO to prepare a 300 mmol/L stock solution. All the stock solutions were aliquoted and stored at −80 °C, with the exception of the NaLa. The concentration of DMSO in the IVC medium was equivalent and less than 0.1% in the different groups. The embryos were continuously cultured in IVC medium with different concentrations of NaLa, GSKA, or NMN after in vitro fertilization.

### Inhibition of DNA replication, RNA synthesis, and protein synthesis using aphidicolin (APD), α-amanitin (α-AMA), and cycloheximide (CHX), respectively

APD (Yuanye, Shanghai, China) was diluted with DMSO to prepare a 3 mg/mL stock solution. APD at 3 μg/mL was used to inhibit DNA replication [18]. α-AMA (MedChemExpress) was diluted with DMSO to prepare a 25 mg/mL stock solution. α-AMA at 25 μg/mL was used to inhibit RNA synthesis [19]. CHX (MedChemExpress) was diluted with DMSO to prepare a 50 mg/mL stock solution. CHX at 50 μg/mL was used to inhibit protein synthesis [20]. All the stock solutions were aliquoted and stored at −80 °C. The concentration of DMSO in the IVC medium was equivalent for and less than 0.1% in the different groups. To inhibit DNA replication, RNA synthesis and protein synthesis, 8-cell embryos were treated with 3 μg/mL APD, 25 μg/mL α-AMA, or 50 μg/mL CHX for 24 h, respectively.

### Immunofluorescence (IF) staining

Embryos were washed with Dulbecco's phosphate-buffered saline (Gibco) supplemented with 0.3% polyvinyl pyrrolidone (DPBS-PVP), fixed with 4% paraformaldehyde and permeabilized with 0.5% Triton X-100 (Solarbio, Shanghai, China) at room temperature. The embryos were then blocked in 2% bovine serum albumin‌ for 1 h at room temperature and incubated with the indicated primary and secondary antibodies. The nuclei were stained with DAPI. Immunofluorescence staining was analyzed using a STELLARIS confocal laser scanning microscope (DMi8; Leica, Wetzlar, Germany). The images were analyzed using ImageJ software (NIH, Bethesda, MD, USA) (https://imagej.net/ij/docs/guide/146-30.html#toc-Subsection-30.2) and based on the methodology described by Ross et al. [21]. In brief, the fluorescence intensity of nucleus was background corrected by dividing by the average of at least three different cytoplasmic areas. The fluorescence levels were quantified as the ratio of nuclear fluorescence intensity to nuclear area. Antibody information is provided in Table S1.

### RNA precursor 5-ethynyluridine (EU) staining assay

Incorporated EU was assayed using a click-iT RNA Alexa Fluor 488 Imaging Kit (Invitrogen, Rochester, NY, USA) in accordance with the manufacturer's instructions. Eight-cell embryos were washed and cultured in prewarmed IVC medium supplemented with 2 mmol/L EU for 1 h. After being washed with DPBS-PVP, the embryos were fixed with 4% paraformaldehyde for 15 min and permeabilized with 0.5% Triton X-100 for 20 min at room temperature. Then, the embryos were transferred to a reaction cocktail for 30 min and subsequently washed with reaction rinse buffer once for 5 min. The nuclei were labelled with Hoechst (10 µg/mL; Invitrogen) for 15 min at room temperature. The embryos were analyzed using a confocal laser scanning microscope (DMi8; Leica) and ImageJ (NIH).

### RNA extraction and quantitative real-time reverse transcription polymerase chain reaction (qRT‒PCR)

Eight-cell embryos were washed with DPBS-PVA three times, and RNA was extracted with a RNeasy Plus Micro Kit (QIAGEN, Dusseldorf, Germany) in accordance with the manufacturer’s instructions. A NanoPhotometer N50 (Implen, München, Germany) was used to assess RNA quality and concentration. RNA with an 260/280 optical density (OD) ratio ranging from 1.8 to 2.0 and 260/230 OD ratio higher than 2.0 was used for subsequent experiments. Total RNA (0.1 μg) was synthesized to first-strand cDNA using a reverse transcription kit (Biosharp, Beijing, China), and equal amounts of RNA input were used across samples. SYBR Green (Biosharp) was used for qRT‒PCR. The qRT‒PCR conditions included denaturation at 95 °C for 3 min and 40 cycles of 95 °C for 10 s, 60 °C for 20 s, and 72 °C for 30 s. Each sample was analyzed with *GAPDH* as the reference gene. mRNA expression was quantified using the 2^−ΔΔCt^ method. The primer information is provided in Table S2.

### Single-cell RNA sequencing (scRNA-Seq)

Eight-cell embryos were washed with PBS (HyClone, Logan, UT, USA) 3 times and treated for 1 min with 1% streptavidin (AMRESCO, Framingham, MA, USA). After the zona pellucida deforms, the embryo is gently aspirated and expelled repeatedly to remove the zona pellucida. RNA was extracted from the samples using single-cell SMART-seq lysis buffer. Total RNA quantity and purity were analyzed with a Thermo Fisher Qubit 3.0 (Thermo Fisher Scientific, Waltham, MA, USA). polyA RNA in the samples was used as the template, and the oligo(dT) sequence with an adapter was used as the primer. Single-strand synthesis was carried out using SMARTScribe™ reverse transcriptase (TaKaRa, Kyoto, Japan). A complete single-stranded cDNA sequence with adapters was synthesized using SMARTer oligonucleotides as a template. Double-stranded cDNA for library construction was generated by PCR amplification using primer-adapters. A SMART-Seq v4 Ultra Low Input RNA Kit (Clontech, Tokyo, Japan) was used to prepare a low-input library. The obtained SMART preamplified products were fragmented by enzymatic digestion. Suitable fragments (150–300 bp) were selected through magnetic bead screening. After the selected fragments were end-repaired and a poly A tail was added to the 3' end, the Y-shaped sequencing adapter was used for ligation. The ligated products were used as templates for PCR amplification to obtain a sequencing library.

Library sequencing was performed on an Illumina NovaSeq 6000 platform (Illumina, San Diego, CA, USA) using a 2 × 150 bp paired-end sequencing protocol. An analysis of differentially expressed genes (DEGs) was performed using edgeR (*P* < 0.05 and absolute fold change ≥ 2). Functional enrichment and pathway enrichment analyses of the DEGs were performed with GO (GeneOntology.org/) [22] and KEGG (https://www.kegg.jp/) [23] analyses.

### Statistical analysis

Statistical analysis was performed using IBM SPSS statistics software (Version 26.0 for Windows; IBM Corp., Armonk, NY, USA). The data were tested for normality before applying parametric statistical tests. The data are presented as the mean ± SEM. The sample size (*n*) of embryos used in each group and biological replicates (*R*) in each experiment are shown in the figure legends. Statistical comparisons of two groups were performed using Student's *t*-test. One-way ANOVA with Tukey’s HSD post hoc multiple comparison procedure was used to compare the data for more than 2 groups. Significant differences are indicated with different letters (*P* < 0.05).

## Results

### Histone lactylation is essential for early embryonic development in bovine

**Fig. 1:**
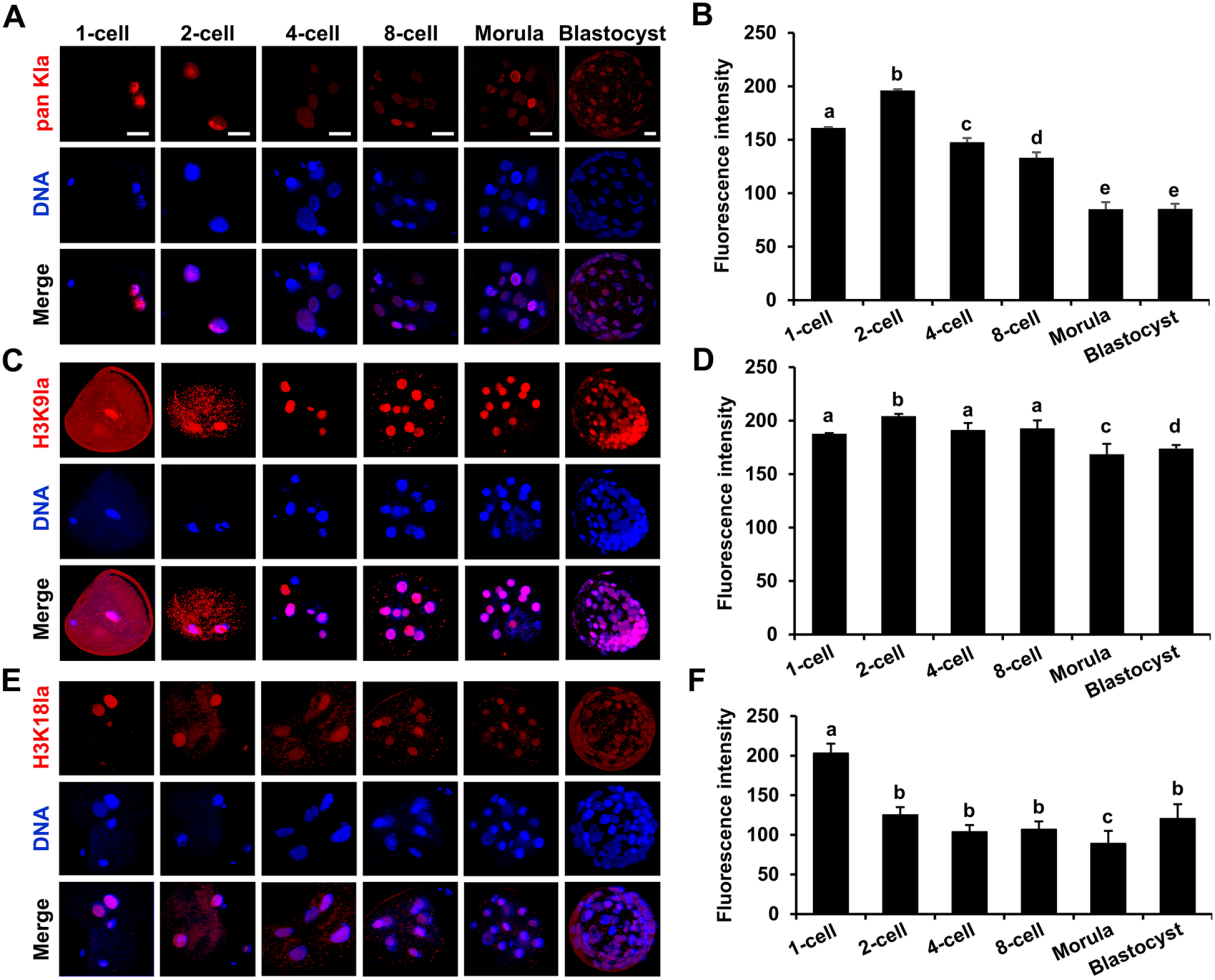
Dynamics of histone lactylation during early bovine embryo development. **A** and **B** Representative images of pan Kla immunofluorescence staining and fluorescence intensity level changes at the 1-cell (*n* = 35), 2-cell (*n* = 24), 4-cell (*n* = 24), 8-cell (*n* = 28), morula (*n* = 32), and blastocyst (*n* = 28) stages of bovine IVF embryos. Embryos were stained for pan Kla (red) and DNA (blue). *R* = 3. Reference scale bar: 25 µm. **C** and **D** Representative images of H3K9la immunofluorescence staining and fluorescence intensity level changes at the 1-cell (*n* = 38), 2-cell (*n* = 31), 4-cell (*n* = 35), 8-cell (*n* = 32), morula (*n* = 38), and blastocyst (*n* = 34) stages of bovine IVF embryos. Embryos were stained for H3K9la (red) and DNA (blue). *R* = 3. Reference scale bar: 25 µm. **E** and **F** Representative images of H3K18la immunofluorescence staining and fluorescence intensity changes of 1-cell (*n* = 38), 2-cell (*n* = 28), 4-cell (*n* = 24), 8-cell (*n* = 25), morula (*n* = 30), and blastocyst (*n* = 34) stages of bovine IVF embryos. Embryos were stained for H3K18la (red) and DNA (blue). *R* = 3. Reference scale bar: 25 µm. One-cell embryo, 2-cell embryo, 4-cell embryo, 8-cell embryo, morula, and blastocyst were collected at 16 h, 30 h, 48 h, 60 h, 108 h, and 168 h post-IVF, respectively. One-way ANOVA with Tukey’s HSD post hoc multiple comparison test was used to compare the data. Different letters on the bars indicate significant differences (*P* < 0.05)

To determine the dynamics of histone lactylation, IF staining was utilized to examine the pan Kla, H3K9la, and H3K18la levels during early bovine embryo development. pan Kla, H3K9la, and H3K18la were present at all stages and were located mainly in the nuclei of bovine IVF embryos (Fig. 1A–F). The pan Kla level significantly increased from the 1-cell stage to the 2-cell stage but gradually decreased from the 2-cell stage to the blastocyst stage (Fig. 1A and B). The H3K9la level was greatest in the 2-cell stage and lowest in the morula stage (Fig. 1C and D). The H3K18la level tended to decrease from the 1-cell stage to the morula stage but significantly increased from the morula stage to the blastocyst stage (Fig. 1E and F). Taken together, these results indicate that pan Kla, H3K9la, and H3K18la modifications are located mainly in the nucleus and that the levels of histone lactylation change dynamically, indicating that these modifications are important for early bovine embryo development.

### Histone lactylation is dependent on DNA replication, RNA synthesis, and protein synthesis

**Fig. 2:**
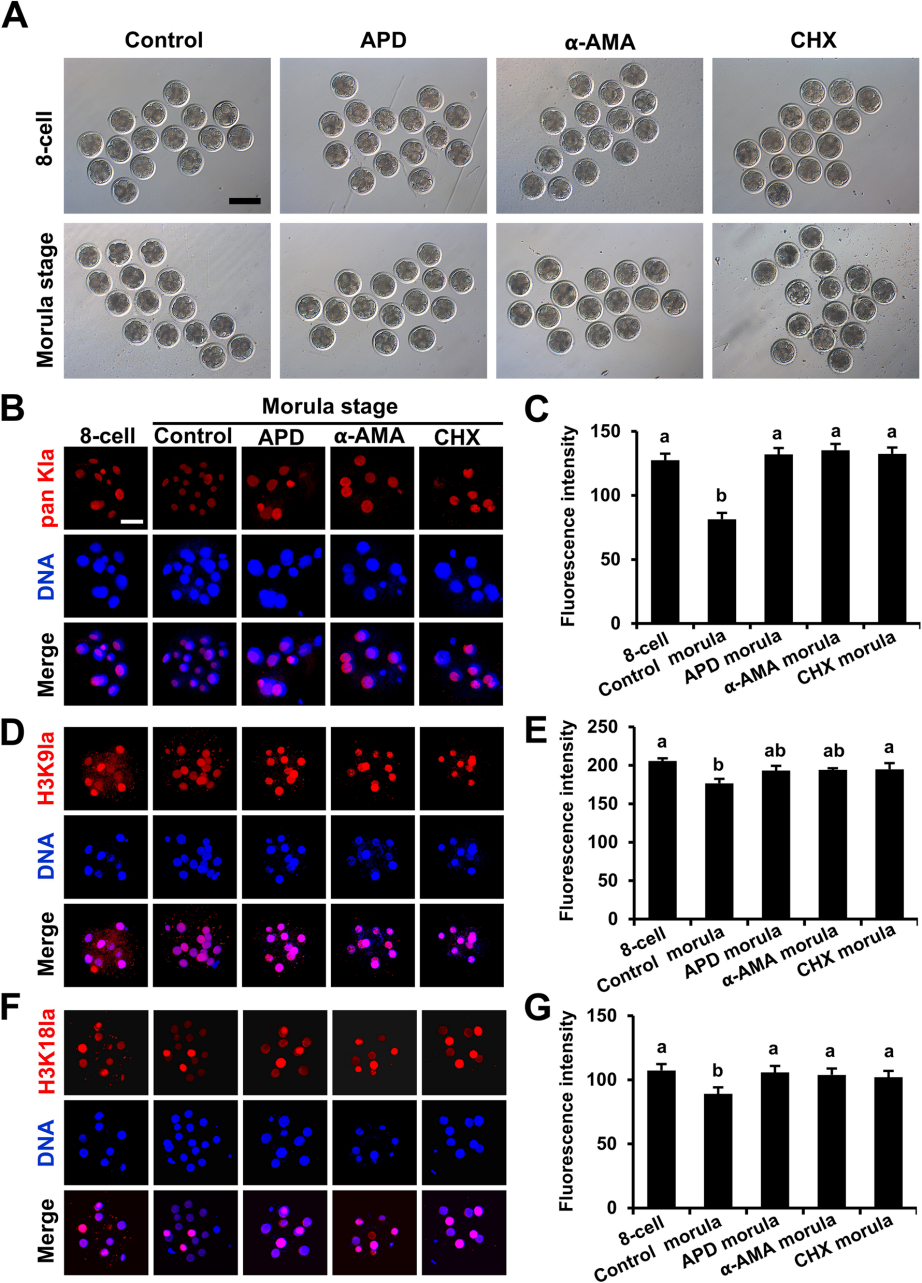
Effects of the inhibition of DNA replication, RNA synthesis, and protein synthesis on histone lactylation levels in embryos. **A** Representative images of 8-cell embryos and morula in the control (*n* = 45), 3 μg/mL APD (*n* = 45), 25 μg/mL α-AMA (*n* = 45), and 50 μg/mL CHX (*n* = 45) groups. Reference scale bar: 200 µm. *R* = 3. Eight-cell embryos were treated with 3 μg/mL APD, 25 μg/mL α-AMA, or 50 μg/mL CHX. 8-cell embryos and morula were imaged and collected at 60 h and 108 h post-IVF, respectively. **B** and **C** Representative images of pan Kla immunofluorescence staining and fluorescence intensity changes in bovine IVF embryos in the 8-cell (*n* = 28), morula (*n* = 26), 3 μg/mL APD-treated morula (*n* = 26), 25 μg/mL α-AMA-treated morula (*n* = 24), and 50 μg/mL CHX-treated morula (*n* = 24) groups. Embryos were stained for pan Kla (red) and DNA (blue). *R* = 3. Reference scale bar: 25 µm. **D** and **E** Representative images of H3K9la immunofluorescence staining of and fluorescence intensity changes in bovine IVF embryos in the 8-cell (*n* = 30), morula (*n* = 30), 3 μg/mL APD-treated morula (*n* = 30), 25 μg/mL α-AMA-treated morula (*n* = 30), and 50 μg/mL CHX-treated morula (*n* = 30) groups. Embryos were stained for H3K9la (red) and DNA (blue). *R* = 3. Reference scale bar: 25 µm. **F** and **G** Representative images of H3K18la immunofluorescence staining of and fluorescence intensity changes in bovine IVF embryos in the 8-cell (*n* = 30), morula (*n* = 29), 3 μg/mL APD-treated morula (*n* = 28), 25 μg/mL α-AMA-treated morula (*n* = 28), and 50 μg/mL CHX-treated morula (*n* = 26) groups. Embryos were stained for H3K18la (red) and DNA (blue). *R* = 3. Reference scale bar: 25 µm. One-way ANOVA with Tukey’s HSD post hoc multiple comparison test was used to compare the data. Different letters on the bars indicate significant differences (*P* < 0.05)

To explore the relationships between histone lactylation modification and the processes of DNA replication, RNA synthesis, and protein synthesis, specific inhibitors were used to inhibit DNA replication, RNA synthesis, and protein synthesis, and the levels of pan Kla, H3K9la, and H3K18la were assessed in early embryos. Our results revealed that in the control group, embryos grew to the morula stage, whereas in the APD, α-AMA, and CHX treatment groups, the embryos arrested at the 8-cell stage (Fig. 2A). All inhibitor treatments prevented the reduction in pan Kla (Fig. 2B and C), H3K9la (Fig. 2D and E), and H3K18la (Fig. 2F and G) modifications from the 8-cell stage to the morula stage. These data demonstrate that histone lactylation is a passive process that is dependent on DNA replication, RNA synthesis, and protein synthesis.

### GSKA and NaLa supplementation inhibits development and lineage differentiation in early bovine embryos

**Fig. 3:**
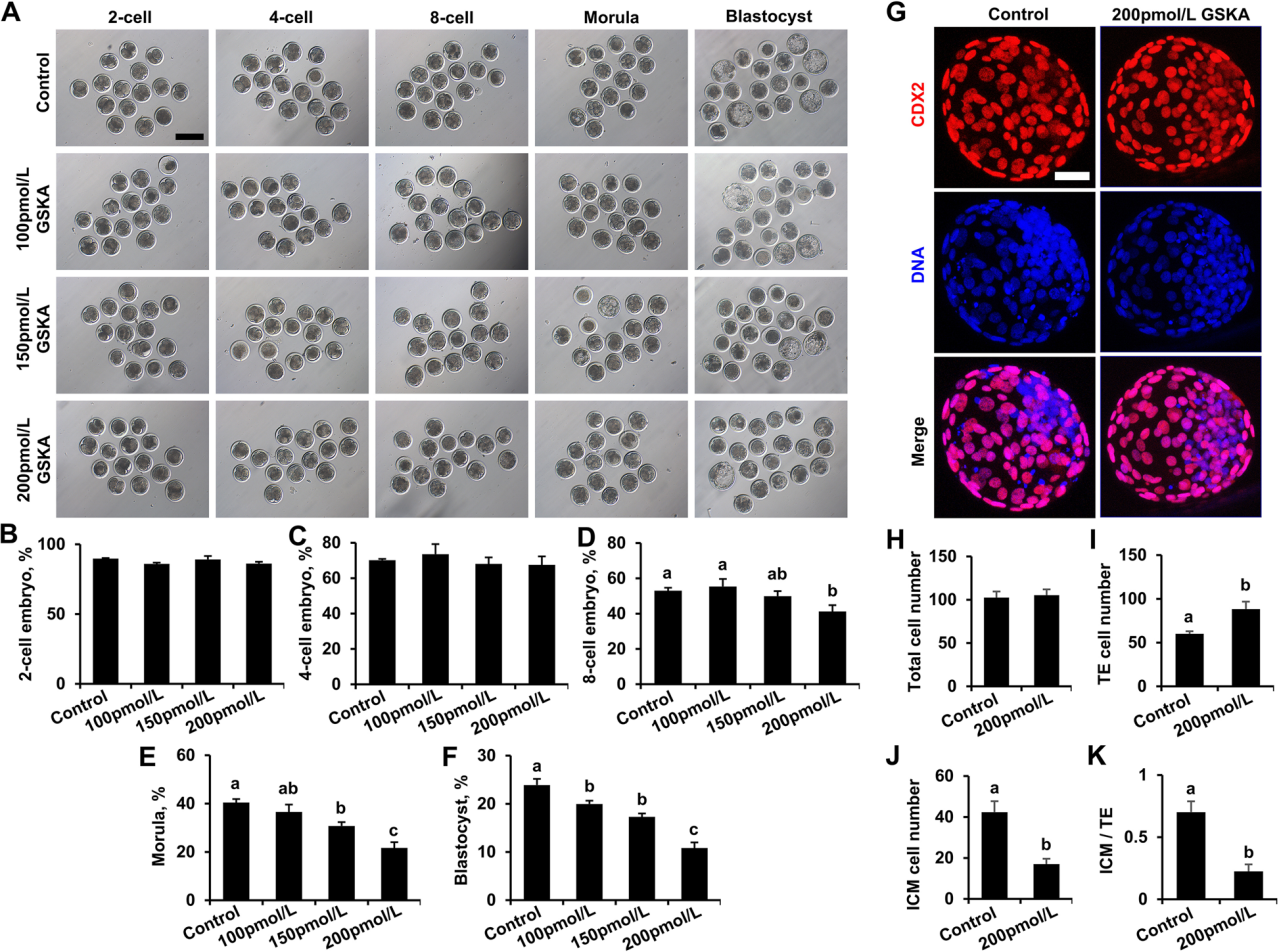
Effects of GSKA supplementation on early bovine embryo development. **A** Representative images of 2-cell embryo, 4-cell embryo, 8-cell embryo, morula, and blastocyst from the control, 100 pmol/L, 150 pmol/L, and 200 pmol/L GSKA groups. Reference scale bar: 200 µm. Two-cell embryo, 4-cell embryo, 8-cell embryo, morula, and blastocyst were imaged at 30 h, 48 h, 60 h, 108 h, and 168 h post-IVF, respectively. The rates of 2-cell embryo (**B**), 4-cell embryo (**C**), 8-cell embryo (**D**), morula (**E**), and blastocyst (**F**) in the control (*n* = 134), 100 pmol/L (*n* = 134), 150 pmol/L (*n* = 134), and 200 pmol/L GSKA (*n* = 132) groups. *R* = 4. **G** Representative images of CDX2 immunofluorescence staining of blastocysts from the control and 200 pmol/L GSKA groups. Reference scale bar: 50 µm. Embryos were stained for CDX2 (red) and DNA (blue). Reference scale bar: 25 µm. The total cell number (**H**), TE cell number (**I**), ICM cell number (**J**), and ICM/TE cell ratio (**K**) for blastocysts from the control (*n* = 26) and 200 pmol/L GSKA (*n* = 26) groups.* R* = 3. Statistical comparisons of the data from Figs. 3B–F were performed using one-way ANOVA with Tukey’s HSD post hoc multiple comparison test. Statistical comparisons of the data from Figs. 3H–K were performed using Student's *t* test. Different letters on the bars indicate significant differences (*P* < 0.05)

GSKA reduces intracellular lactate production by inhibiting lactate dehydrogenase A (LDHA) activity, thereby decreasing the level of histone lactylation. NaLa, as an exogenous lactate donor, increases the level of histone lactylation by increasing the intracellular lactate concentration. To explore the effects of histone lactylation modification on embryo development and lineage differentiation, the embryo development rate and inner cell mass (ICM)/trophectoderm (TE) ratio were assessed after treatment with different concentrations of GSKA or NaLa. The results revealed that there was no significant difference in the rate of 2-cell or 4-cell embryo formation between the GSKA treatment group and the control group (Fig. 3A–C). Compared with that in the control group, the rate of 8-cell embryo formation significantly decreased after treatment with 200 pmol/L GSKA (41.89% ± 3.54% vs. 52.25% ± 1.42%; *P* < 0.01; Fig. 3A and D), and the rate of morula and blastocyst formation significantly decreased after treatment with 150 pmol/L and 200 pmol/L GSKA (Fig. 3A, E, and F). The blastocyst formation rates in the control and 200 pmol/L GSKA groups were 23.76% ± 0.93% and 11.46% ± 1.04%, respectively (*P* < 0.01). Therefore, a concentration of 200 pmol/L was selected for subsequent experiments.

To determine the effect of histone lactylation on embryo lineage differentiation, IF staining was used to evaluate caudal-type homeobox 2 (CDX2) and DNA levels in blastocysts after treatment with 200 pmol/L GSKA. We found that there was no significant difference in the total number of blastocysts between the 200 pmol/L GSKA group and the control group. However, compared with those in the control group, the TE cell number was significantly higher, the ICM cell number was significantly lower, and the ICM/TE ratio was significantly lower in the 200 pmol/L GSKA group (Fig. 3G–K). Therefore, these results show that GSKA treatment inhibits early embryonic development and blastocyst lineage differentiation.

We also showed that there was no significant difference in the rate of 2-cell embryo between the NaLa group and the control group (Fig. S1A–C). Compared with those in the control group, the rates of 4-cell embryo, morula, and blastocyst were significantly lower in the 5 mmol/L NaLa group (Fig. S1A, C, E, and F), and the rates of 8-cell embryos, morula and blastocyst were significantly lower in the 10 mmol/L NaLa group (Fig. S1A, D, E, and F). Therefore, a concentration of 10 mmol/L was selected to evaluate the effects of NaLa on the ICM/TE ratio of blastocysts. We also found that there was no significant difference in the total cell number of blastocysts between the 10 mmol/L NaLa group and the control group. However, compared with those in the control group, the TE cell number was significantly higher, the ICM cell number was significantly lower, and the ICM/TE ratio was significantly lower in the 10 mmol/L NaLa group (Fig. 3G–K). Therefore, these results show that NaLa treatment inhibits early embryonic development and blastocyst lineage differentiation.

Together, these data indicate that GSKA and NaLa inhibit early bovine embryo development and blastocyst lineage differentiation.

### GSKA and NaLa supplementation alters histone lactylation levels in early bovine embryos

**Fig. 4:**
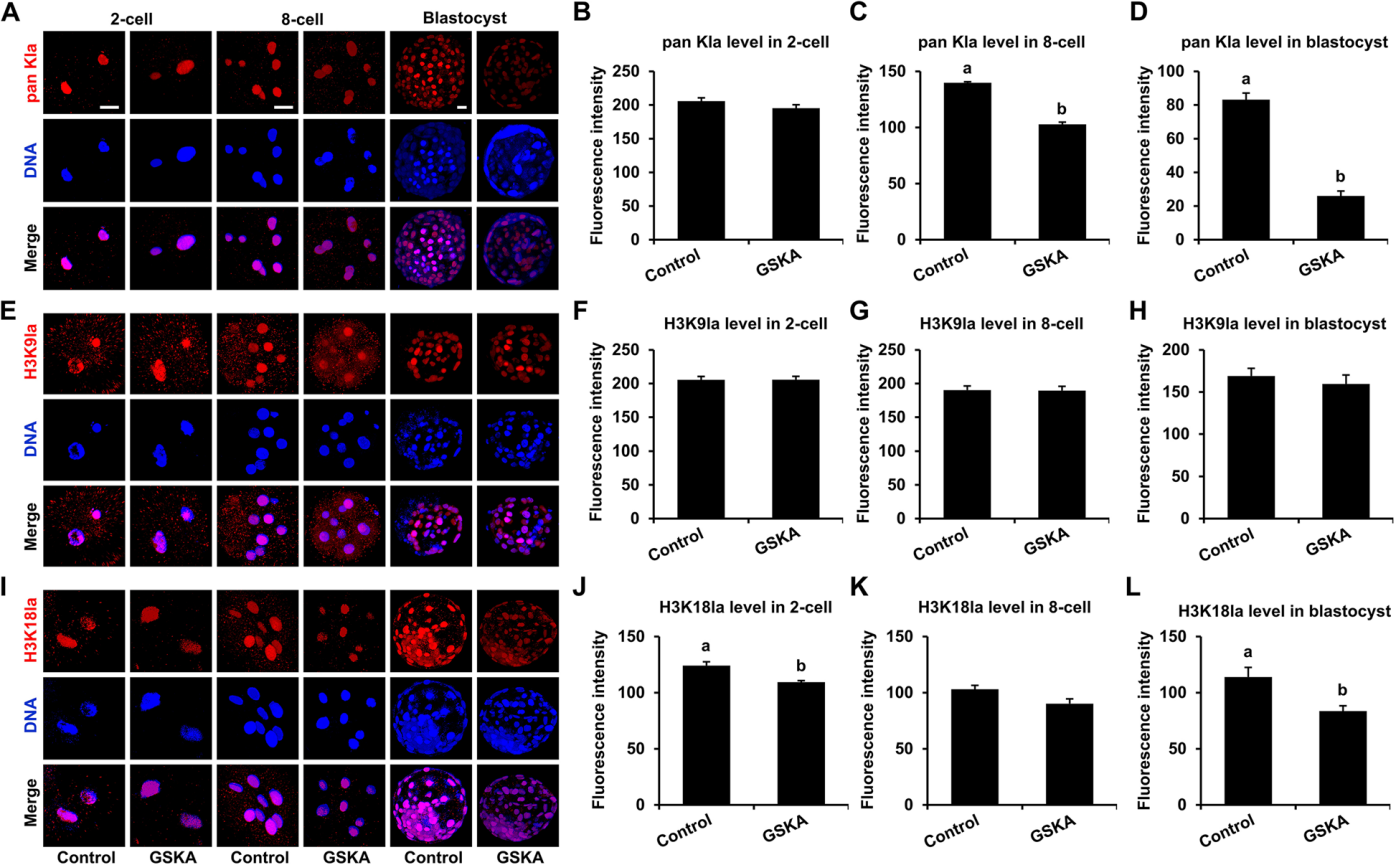
Effects of GSKA supplementation on histone lactylation modification level in bovine embryo. **A** Representative images of pan Kla immunofluorescence staining of 2-cell embryo, 8-cell embryo, and blastocyst from the control and 200 pmol/L GSKA groups. Embryos were stained for pan Kla (red) and DNA (blue). Reference scale bar: 25 µm. Two-cell embryos, 8-cell embryos, and blastocysts were collected at 30 h, 60 h, and 168 h post-IVF, respectively. **B** The pan Kla fluorescence intensity level changes of 2-cell embryo in the control (*n* = 24) and 200 pmol/L GSKA (*n* = 25) groups. *R* = 3. **C** The pan Kla fluorescence intensity level changes of 8-cell embryo in the control (*n* = 25) and 200 pmol/L GSKA (*n* = 24) groups. *R* = 3. **D** The pan Kla fluorescence intensity level changes of blastocyst in the control (*n* = 24) and 200 pmol/L GSKA treatment (*n* = 24) groups. *R* = 3. **E** Representative images of H3K9la immunofluorescence staining of 2-cell embryo, 8-cell embryo, and blastocyst from the control and 200 pmol/L GSKA groups. Embryos were stained for H3K9la (red) and DNA (blue). Reference scale bar: 25 µm. **F** The H3K9la fluorescence intensity level changes of 2-cell embryo in the control (*n* = 26) and 200 pmol/L GSKA (*n* = 28) groups. *R* = 3. **G** The H3K9la fluorescence intensity level changes of 8-cell embryo in the control (*n* = 25) and 200 pmol/L GSKA (*n* = 24) groups. *R* = 3. **H** The H3K9la fluorescence intensity level changes of blastocyst in the control (*n* = 25) and 200 pmol/L GSKA (*n* = 26) groups. *R* = 3. **I** Representative images of H3K18la immunofluorescence staining of 2-cell embryo, 8-cell embryo, and blastocyst in the control and 200 pmol/L GSKA groups, respectively. Embryos were stained for H3K18la (red) and DNA (blue). Reference scale bar: 25 µm. **J** The H3K18la fluorescence intensity level changes of 2-cell embryo in the control (*n* = 26) and 200 pmol/L GSKA (*n* = 25) groups. *R* = 3. **K** The H3K18la fluorescence intensity level changes of 8-cell embryo in the control (*n* = 28) and 200 pmol/L GSKA (*n* = 28) groups. *R* = 3. **L** The H3K18la fluorescence intensity level changes of blastocyst in the control (*n* = 27) and 200 pmol/L GSKA (*n* = 25) groups. *R* = 3. Statistical comparisons were performed using Student's *t*-test. Different letters on the bars indicate significant differences (*P* < 0.05)

To further examine the effects of GSKA and NaLa on histone lactylation and acetylation, IF staining was performed to evaluate the pan Kla, H3K9la, H3K18la, histone H3 lysine 9 acetylation (H3K9ac), and histone H3 lysine 27 acetylation (H3K27ac) levels during early bovine embryo development. Our results revealed that compared with those in the control group, the pan Kla levels in 2-cell embryos were not significantly different whereas those in 8-cell embryos and blastocysts were significantly lower in the 200 pmol/L GSKA group (Fig. 4A and B). There was no significant difference in H3K9la levels at the 2-cell, 4-cell, or blastocyst stage between the control group and the 200 pmol/L GSKA group (Fig. 4C and D). Compared with those in the control group, H3K18la levels in 8-cell embryos were not significantly different whereas those in 2-cell embryos and blastocysts were significantly lower in the 200 pmol/L GSKA group (Fig. 4E and F).

We also found that compared with those in the control group, the pan Kla levels in 2-cell embryos were not significantly different whereas those in 8-cell embryos and blastocysts were significantly higher in the 10 mmol/L NaLa group (Fig. S2A and B). There was no significant difference in H3K9la levels at the 2-cell or 4-cell stage between the control group and the 10 mmol/L NaLa group. However, the H3K9la level in blastocysts increased significantly after treatment with 10 mmol/L NaLa (Fig. S2C and D). Compared with those in the control group, H3K18la levels in 2-cell embryos, 4-cell embryos, and blastocysts were significantly higher in the 10 mmol/L NaLa group (Fig. S2E and F). In addition, our study revealed that there was no significant difference in H3K9ac and H3K27ac levels at the 2-cell, 4-cell, and blastocyst stages among the control, 200 pmol/L GSKA, and 10 mmol/L NaLa groups (Fig. S3 and S4). Together, these results indicate that GSKA and NaLa affect embryo development by regulating histone lactylation rather than acetylation.

### Histone lactylation affects the transcriptional activity of the bovine embryonic genome

**Fig. 5:**
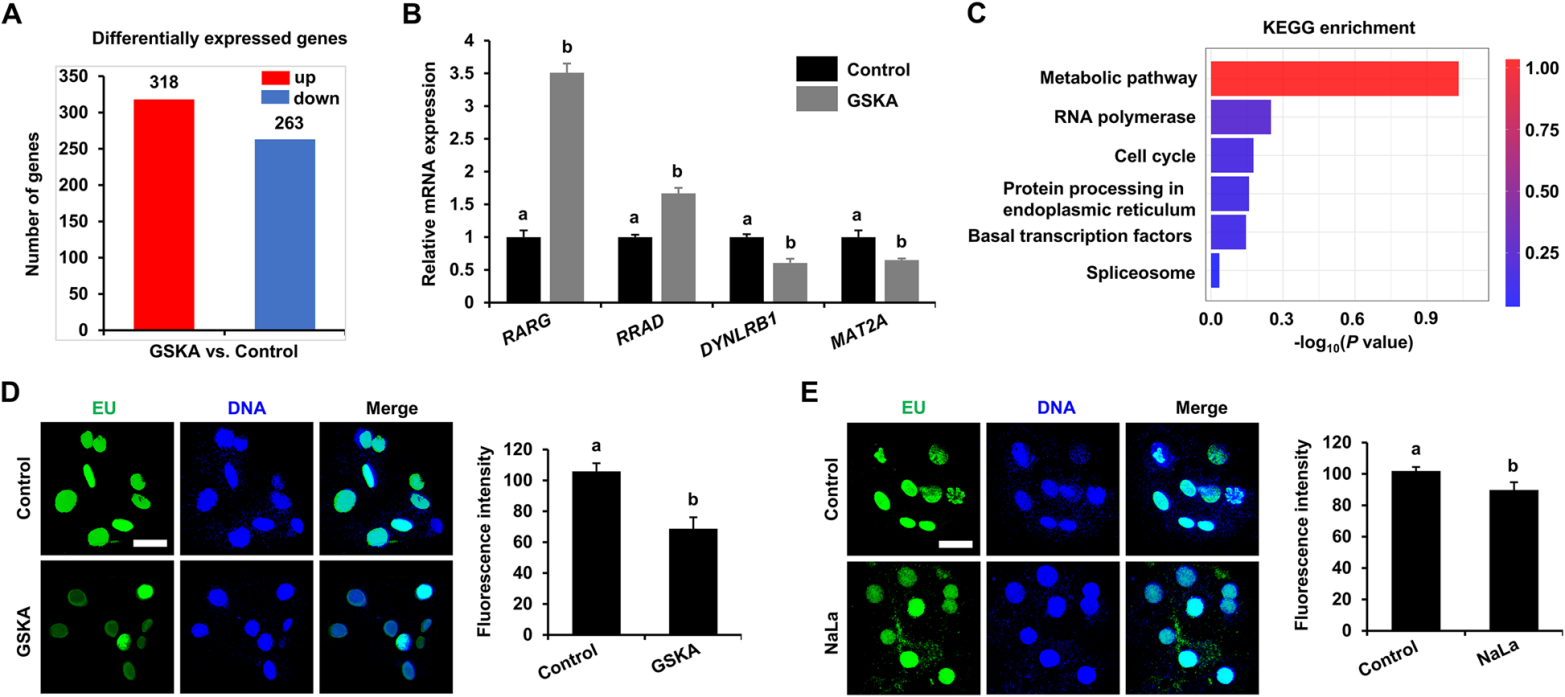
Effects of GSKA on genomic transcriptional activity in bovine early embryo. **A** DEGs by scRNA-seq analysis after 200 pmol/L GSKA treatment in bovine 8-cell embryo. Red bar represents upregulated DEGs, with a total of 318 genes; blue bar represents downregulated DEGs, with a total of 263 genes. *R* = 3. **B** Relative mRNA expression level changes of *RARG*, *RRAD*, *DYNLRB1*, and *MAT2A* using qRT-PCR of 8-cell embryo in the control (*n* = 240) and 200 pmol/L GSKA (*n* = 240) groups. *R* = 3. Data were compared using the Student's *t*-test. **C** KEGG pathway enrichment of DEGs. The length and color of bars in the figure represents −log_10_(*P* value). **D** Representative images of EU staining and fluorescence intensity level changes of 8-cell embryo in the control (*n* = 28) and 200 pmol/L GSKA treatment (*n* = 27) groups. *R* = 3. Embryos were stained for EU (green) and DNA (blue). Reference scale bar: 25 µm. Data were compared using the Student's *t*-test. **E** Representative images of EU staining and fluorescence intensity level changes of 8-cell embryo from the control (*n* = 28) and 10 mmol/L NaLa (*n* = 25) groups. *R* = 3. Embryos were stained for EU (green) and DNA (blue). Reference scale bar: 25 µm. Data were compared using the Student's *t*-test. Different letters on the bars indicate significant differences (*P* < 0.05)

To further investigate the molecular mechanism through which histone lactylation regulates embryonic development, scRNA-seq was used to evaluate the transcriptomic changes in 8-cell-stage bovine embryos after treatment with 200 pmol/L GSKA. Principal component analysis (PCA) indicated that the samples presented good intragroup clustering, and significant differences were observed between the control and GSKA groups (Fig. S5A). A total of 581 DEGs were identified between the control and GSKA groups, of which there were 318 upregulated genes and 263 downregulated genes (Fig. 5A, S5B and C; Table S3). To validate the scRNA-seq data, the expression levels of 4 DEGs (2 upregulated and 2 downregulated genes), namely, retinoic acid receptor gamma (*RARG*), Ras-related glycolysis inhibitor and calcium channel regulator (*RRAD*), dynein light chain roadblock-type 1 (*DYNLRB1*), and methionine adenosyltransferase 2 A (*MAT2A*), were analyzed by qRT‒PCR. The expression patterns of these genes were consistent with the trends observed in the scRNA-seq data (Fig. 5B and Table S3), confirming the robustness of the scRNA-seq data. GO enrichment analysis revealed that the DEGs were enriched mainly in the regulation of DNA-templated transcription, the regulation of transcription by RNA polymerase II, the nucleus, protein binding, DNA binding, etc. (Fig. S5D). KEGG enrichment analysis revealed that the DEGs were enriched mainly in metabolic pathways, RNA polymerase, the cell cycle, protein processing in the endoplasmic reticulum, basal transcription factors, and the spliceosome (Fig. 5B). Moreover, compared with that in the control group, the EU levels were significantly lower in the GSKA and NaLa groups (Fig. 5D and E). Therefore, these data demonstrate that abnormal histone lactylation decreases the transcriptional activity of the bovine embryonic genome.

### NMN rescues the inhibitory effect of GSKA supplementation on early bovine embryo development

**Fig. 6:**
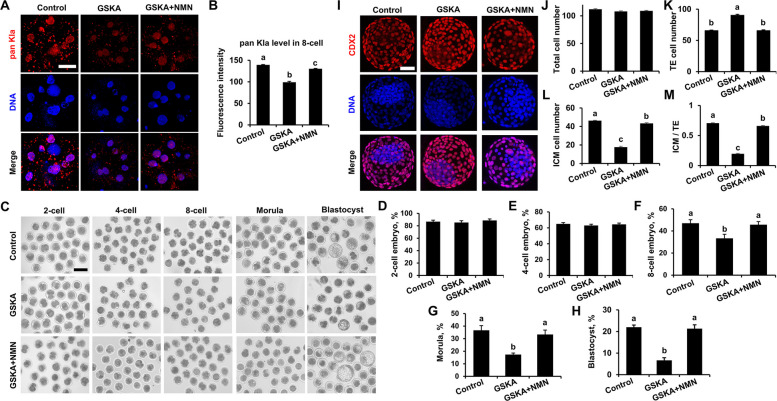
Effects of NMN on bovine early embryo development affected by GSKA. **A **and** B** Representative images of pan Kla immunofluorescence staining and fluorescence intensity level changes of 8-cell embryo in the control (*n* = 9), 200 pmol/L GSKA (*n* = 9), and 200 pmol/L GSKA + 300 μmol/L NMN (*n* = 9) groups. *R* = 3. Embryos were stained for pan Kla (red) and DNA (blue). Reference scale bar: 25 µm. **C** Representative images of 2-cell embryo, 4-cell embryo, 8-cell embryo, morula, and blastocyst in the control, 200 pmol/L GSKA, and 200 pmol/L GSKA + 300 μmol/L NMN groups, respectively. Reference scale bar: 200 µm. Two-cell embryo, 4-cell embryo, 8-cell embryo, morula, and blastocyst were imaged at 30 h, 48 h, 60 h, 108 h, and 168 h post-IVF, respectively. The rates of 2-cell embryo (**D**), 4-cell embryo (**E**), 8-cell embryo (**F**), morula (**G**), and blastocyst (**H**) in the control (*n* = 150), 200 pmol/L GSKA treatment (*n* = 150), and 200 pmol/L GSKA + 300 μmol/L NMN treatment (*n* = 150) groups. *R* = 3. (**I**) Representative images of CDX2 immunofluorescence staining of blastocyst in the control, 200 pmol/L GSKA, and 200 pmol/L GSKA + 300 μmol/L NMN groups. *R* = 3. Reference scale bar: 50 µm. Embryos were stained for CDX2 (red) and DNA (blue). Reference scale bar: 25 µm. The total cell number (**J**), TE cell number (**K**), ICM cell number (**L**), and ICM/TE cell ratio (**M**) of blastocyst in control (*n* = 19), 200 pmol/L GSKA (*n* = 11), and 200 pmol/L GSKA + 300 μmol/L NMN (*n* = 16) groups. *R* = 3. Statistical comparisons were performed using one-way ANOVA with Tukey’s HSD post hoc multiple comparison procedure. Different letters on the bars indicate significant differences (*P* < 0.05)

NMN increases the lactylation level by providing nicotinamide adenine dinucleotide (NAD^+^). To explore the effects of NMN on early bovine embryo development, which is affected by reduced levels of histone lactylation, embryo development efficiency and blastocyst lineage differentiation were assessed after cotreatment with 200 pmol/L GSKA and 300 μmol/L NMN. We found that NMN rescued the reduction in pan Kla level affected by GSKA treatment. However, the pan Kla level in the GSKA + NMN group remained significantly lower, compared with those in the control group (Fig. 6A and B). There was no significant difference in rate of 2-cell or 4-cell embryo formation among the control, GSKA, and GSKA + NMN groups (Fig. 6C–E). Compared with the control group, treatment with GSKA significantly decreased the 8-cell embryo, morula, and blastocyst formation rates, whereas cotreatment with GSKA and NMN reversed these inhibitory effects and increased the 8-cell embryo, morula, and blastocyst formation rates. The rates of 8-cell embryo formation in the control, GSKA, and GSKA + NMN groups were 47.03% ± 3.16%, 27.33% ± 0.67%, and 45.67% ± 2.77%, respectively. The blastocyst formation rates in the control, GSKA, and GSKA + NMN groups were 22.81% ± 1.14%, 6.64% ± 1.23%, and 21.19% ± 1.49%, respectively. There was no significant difference in rate of 8-cell embryo, morula, or blastocyst formation between the GSKA + NMN group and the control group (Fig. 6C and F–H). Compared with those in the GSKA group, the TE cell number were significantly lower, ICM cell number and ICM/TE rate of blastocysts were significantly higher in the GSKA + NMN group. There was no significant difference in TE cell number between the GSKA + NMN group and the control group, but compared with those in the control group, the ICM cell number and ICM/TE ratio in the GSKA + NMN group remained significantly lower (Fig. 6I–M). Hence, these results indicate that NMN rescues the inhibitory effect of reduced histone lactylation on early bovine embryo development.

## Discussion

Early embryo loss and pregnancy failure are among the most challenging problems faced by the cattle industry [24]. The key to resolving this problem lies in the smooth progression of various biological events during the embryonic development process, such as EGA. Histone lysine lactylation, a newly discovered epigenetic modification that links cellular metabolism with epigenetic regulation, affects early embryonic development by regulating EGA [25, 26]. This study revealed that histone lactylation dynamically changes during early embryonic development and that abnormal histone lactylation inhibits the transcriptional activity of embryonic genome, early embryonic development, and blastocyst lineage differentiation in bovine. Our findings indicate that histone lactylation plays crucial roles in early bovine embryo development through the regulation of EGA.

After fertilization, the embryo undergoes a series of epigenetic reprogramming events that lead to genome activation and lineage differentiation, thereby enabling the embryo to acquire totipotency [27, 28]. Our results revealed that in bovine IVF embryos, pan Kla, H3K9la, and H3K18la levels significantly decreased from the 8-cell stage to the morula stage; additionally, H3K9la and H3K18la levels significantly increased and pan Kla levels tended to increase from the morula stage to the blastocyst stage. EGA and morula-to-blastocyst transition, in which the embryo undergoes differentiation to the TE and ICM, are two main events from the zygote stage to the blastocyst stage during preimplantation embryonic development. Previous studies have shown that epigenetic modifications, such as N^6^-methyladenosine and N^4^-acetylcytidine modifications, are essential for early embryonic development because they regulate these two events [29–32]. These findings indicate that the removal of histone lactylation is required for the EGA process in 8-cell embryos, whereas subsequently, histone lactylation is needed for blastocyst lineage differentiation and embryonic pluripotency [25], suggesting that histone lactylation is crucial for both EGA and the morula-to-blastocyst transition process during early embryonic development in bovine. Interestingly, we found that asymmetric histone lactylation is remodeled post fertilization in cattle. After fertilization, the male pronucleus undergoes intense histone protamine replacement and extensive demethylation, while the female pronucleus retains most of the chromatin characteristics of the oocyte, resulting in asymmetric epigenetic reprogramming, such as H3K9me2, H3K27me3, H3K9ac, H3K18ac, and H3K23ac, between male and female pronuclei in mammals [33–35]. However, a previous study revealed no asymmetry in the levels of histone lactylation modifications, such as pan Kla, H3K18la, and H3K23la, between male and female pronuclei in mice [14]. Differences in lactate metabolism [36], lactate transport efficiency [37], and lactylation-modifying enzyme activity [38, 39] may exist between male and female pronuclei across different species, contributing to the heterogeneity in lactylation modifications.

Furthermore, our research revealed that inhibitors of DNA replication, RNA synthesis, and protein synthesis can significantly inhibit the removal of pan Kla, H3K9la, and H3K18la from the 8-cell stage to the morula stage; therefore, embryo development is arrested at the 8-cell stage. These findings indicate that the epigenetic remodeling of histone lactylation is indispensable for EGA. Additionally, after the disruption of transcriptional and translational processes, the expression of key factors involved in energy metabolism, lactate substrate generation, transport, and lactylation modification was inhibited. That inhibition impaired cellular metabolism and histone lactylation, thereby inhibiting EGA in embryos. Given that pan Kla, H3K9la, and H3K18la occur mainly within the nuclei of early bovine embryos, the aforementioned results indicate that histone lactylation is dependent on DNA replication, RNA synthesis, and protein synthesis during the process of EGA during early embryonic development.

Lactic acid is an important substrate and signaling molecule for energy metabolism and histone lactylation [40]. We found that GSKA supplementation significantly reduced the 8-cell embryo formation rate and blastocyst lineage differentiation by inhibiting lactate production and that the exogenous addition of NaLa resulted in similar effects. These consistent to previous studies, a deficiency of lactic acid in culture medium leads to a decrease in the H3K18la level, 2-cell embryo development arrest, and the abnormal expression of EGA genes in mice [41]. Interestingly, our results revealed that the increase in lactic acid caused by NaLa supplementation inhibited bovine IVF embryo development. A previous study revealed that lactate supplementation increased the developmental potential and blastocyst quality of bovine SCNT embryos; compared with IVF embryos, SCNT embryos had lower LDHA levels and lactate contents, and the addition of lactate significantly increased embryonic development via pan Kla and H3K18la as well as the expression levels of the EGA genes *ZSCAN5B* and *SUPT4H1* [42]. These results indicate that the inhibition of histone lactylation by reduced levels of lactic acid may affect EGA by regulating pan Kla and H3K18la modifications, thereby influencing early embryonic development and lineage differentiation. However, abnormal histone lactylation levels caused by the excessive accumulation of lactate also inhibit embryonic development.

However, neither GSKA nor NaLa significantly changed the bovine 2-cell embryo formation rate. The 2-cell stage is the main period for EGA in mice, while the change in the bovine 2-cell formation rate is related mainly to the successful occurrence of mitosis [43], indicating that histone lactylation has no significant effect on mitosis during the development process of bovine embryos. Interestingly, GSKA and NaLa did not affect H3K9la levels in 8-cell embryos, indicating that H3K9la modification may be weakly dependent on substrates during the reprogramming process. Both lactylation and acetylation occur on histone lysine residues, with highly overlapping modification sites. We revealed that GSKA and NaLa do not affect the levels of H3K9ac and H3K27ac modifications in bovine 2-cell embryos, 8-cell embryos, and blastocysts. These findings indicate that endogenous lactate depletion and exogenous lactate supplementation may not specifically affect P300/CBP or the substrate preference of ACSS2 between acetyl-CoA and lactyl-CoA [41].

Furthermore, we found that the indirect increase in lactate levels induced by NMN supplementation can significantly alleviate the reduction in pan Kla modification and 8-cell embryo, morula, and blastocyst formation rates, as well as the effect of GSKA on embryonic lineage differentiation ability [44]. NMN can be converted into NAD^+^ by NMN adenylyltransferase, providing the essential “molecular fuel” for subsequent lactylation processes [17]. Previous studies have shown that NMN improves bovine oocyte quality and embryo developmental competence through the enhancement of mitochondrial function [45, 46]. NMN can also decrease the level of oxidative stress and apoptosis, thereby improving oocyte maturation and embryo development [47, 48]. Additionally, NAD deficiency is among the many known causes of adverse pregnancy outcomes because it inhibits embryo development [49]. These findings indicate that histone lactylation may maintain the EGA process by regulating mitochondrial function and antioxidant capacity and inhibiting apoptosis in 8-cell embryos, thereby maintaining subsequent embryonic development and lineage differentiation. Moreover, NAD^+^ simultaneously increases mitochondrial oxidative metabolism and partially suppresses glycolysis [50]. A previous study indicated that the inhibition of glycolytic activity decreases intracellular lactate levels under hypoxic conditions [14], thereby inhibiting histone lactylation. These results indicate that NAD⁺ is a critical molecule that tightly links the lactylation process to cellular energy metabolism.

Genomic transcriptional activity is an important indicator of whether EGA has occurred successfully [51]. *MAT2A*, an important RNA polymerase, can regulate transcriptional activity and thereby affect embryonic genome activation in pigs and mice [52]. Our research revealed that GSKA can significantly inhibit the mRNA expression level of *MAT2A* and that both GSKA and NaLa lead to a decreased level of EU [53] fluorescence intensity in 8-cell embryos. The results of the scRNA-seq analysis revealed that DNA binding, DNA-template transcription, RNA polymerase, the cell cycle, and the regulation of transcription by RNA polymerase II are crucial for the EGA process, suggesting that histone lactylation may affect genomic transcriptional activity by regulating DNA binding and RNA polymerase activity [26]. Previous studies have shown that EGA and chromatin remodeling are likely interdependent. The inhibition of embryonic transcription pervasively disrupts the establishment of open chromatin during EGA [54], which indicates that histone lactylation may be essential for chromatin remodeling during the EGA process through the regulation of genomic transcription, thereby maintaining subsequent embryonic development. Notably, compared with the microenvironment within the reproductive tract of cows, the oocyte IVM and embryo IVC environments exhibit distinct differences in terms of oxygen tension, glycolysis concentration, lactic acid concentration, energy metabolism, and lactate availability [10, 14, 41, 55]. These differences affect histone lactylation by altering the lactate precursor supply, enzyme activity, and chromatin state [25, 56].

## Conclusion

In conclusion, the results demonstrate that histone lactylation is required for early bovine embryo development through the regulation of EGA. Our findings provide new insights into the regulatory mechanism of histone lactylation and provide a theoretical basis for analyzing the processes and mechanisms of bovine embryo development.

## Supplementary Information

Additional file 1: Table S1. Antibodies information in this study.

Additional file 2: Table S2. Primer sequences used in this study for qRT-PCR.

Additional file 3: Table S3. DEGs information from scRNA-Seq analysis.

Additional file 4: Fig. S1. Effects of NaLa on bovine early embryo development; Fig. S2. Effects of NaLa on histone lactylation modification level in bovine embryo; Fig. S3. Effects of GSKA and NaLa on H3K9ac levels in bovine early embryo; Fig. S4. Effects of GSKA and NaLa on H3K27ac levels in bovine early embryo; Fig. S5. Differential gene expression profile mediated by reduced histone lactylation levels in early bovine embryos.

## Data Availability

All data generated or analyzed during this study are included in this published article, its supplementary information files, and NCBI GEO repository under accession number GSE315024.

## Abbreviations

α-AMA: α-Amanitin
APD: Aphidicolin
CDX2: Caudal type homeobox 2
CHX: Cycloheximide
DEG: Differentially expressed gene
DPBS-PVP: Dulbecco's phosphate-buffered saline containing polyvinyl pyrrolidone
DYNLRB1: Dynein light chain roadblock-type 1
EGA: Embryonic genome activation
EU: 5-Ethynyluridine
H3K18la: Histone H3 lysine 18 lactylation
H3K27ac: Histone H3 lysine 27 acetylation
H3K9ac: Histone H3 lysine 9 acetylation
H3K9la: Histone H3 lysine 9 lactylation
ICM: Inner cell mass
IF: Immunofluorescence
IVC: In vitro culture
IVF: In vitro fertilization
IVM: In vitro maturation
MAT2A: Methionine adenosyltransferase 2A
NaLa: Sodium lactate
NMN: β-Nicotinamide mononucleotide
pan Kla: Pan-lysine lactylation
PCA: Principal components analysis
qRT-PCR: Quantitative real-time reverse transcription polymerase chain reaction
RARG: Retinoic acid receptor gamma
RRAD: Ras related glycolysis inhibitor and calcium channel regulator
scRNA-Seq: Single-cell RNA sequencing
TE: Trophectoderm
