# Supplementary material for: Histone lactylation maintains bovine early embryo development via regulating embryonic genome activation
Source: J Anim Sci Biotechnol. 2026 Apr 23;17:76. doi: 10.1186/s40104-026-01398-8 (PMC13104507; doi:10.1186/s40104-026-01398-8)
Supplement: Supplementary file 1 — Additional file 1: Table S1. Antibodies information in this study. [file 40104_2026_1398_MOESM1_ESM.docx]

**Supplementary** **Table S1.** Antibodies information in this study

| **Antibody** | **Cat. Number and dilution** | **Company^a^** |
| --- | --- | --- |
| pan Kla | PTM-1401 (1:200) | PTM Bio |
| H3K9la | PTM-1419RM (1:200) | PTM Bio |
| H3K18la | PTM-1406RM (1:200) | PTM Bio |
| RNA Alexa Flour | C10329 | Invitrogen |
| Alexa Fluor 594 anti-rabbit IgG | A11012 (1:200) | Invitrogen |

**^a^** PTM Bio, Chicago, USA.

Invitrogen, Rochester, NY, USA.
