# Supplementary material for: Histone lactylation maintains bovine early embryo development via regulating embryonic genome activation
Source: J Anim Sci Biotechnol. 2026 Apr 23;17:76. doi: 10.1186/s40104-026-01398-8 (PMC13104507; doi:10.1186/s40104-026-01398-8)
Supplement: Supplementary file 2 — Additional file 2: Table S2. Primer sequences used in this study for qRT-PCR. [file 40104_2026_1398_MOESM2_ESM.docx]

**Supplementary Table S2.** Primer sequences used in this study for qRT-PCR

| **Genes^a^** | **Accession number** | | **Primer Sequence (5' to 3') ^a^** | **Product length**  **(bp)** |  |  |
| --- | --- | --- | --- | --- | --- | --- |
| *GAPDH* | | NM_001034034.2 | | F:AAAGTGGACATCGTCGCCATC | 186 |  |
|  |  |  |  | R:ACCCCACTTGATGTTGGCAG |  |  |
| *MAT2A* | | | NM_001101131.1 | F:TCCCTTTTCTCCTCTACTCCACT | | 149 |
|  |  |  |  | R:TGGTCACAGATCTTATCTGGGTG | |  |
| *RARG* | | | NM_001130756.1 | F:GTCTCCACCTTTCGAGATGCT | | 93 |
|  |  |  |  | R:TGTGTCTCCACCGACAGAG | |  |
| *DYNLRB1* | | | NM_001034442.2 | F:GATCGGTTCGAGATGGCAGA | | 124 |
|  |  |  |  | R:TGGGATTGTCCATGGTGCTC | |  |
| *RRAD* | | | NM_001045913.2 | F:TGACATTTGGGAGCAGGATGG | | 107 |
|  |  |  |  | R:TCTCGAAGCTTCCCTTGTCG | |  |

^a^ F: Forward primer; R: Reverse primer.
