# Supplementary material for: Histone lactylation maintains bovine early embryo development via regulating embryonic genome activation
Source: J Anim Sci Biotechnol. 2026 Apr 23;17:76. doi: 10.1186/s40104-026-01398-8 (PMC13104507; doi:10.1186/s40104-026-01398-8)
Supplement: Supplementary file 4 — Additional file 4: Fig. S1. Effects of NaLa on bovine early embryo development; Fig. S2. Effects of NaLa on histone lactylation modification level in bovine embryo; Fig. S3. Effects of GSKA and NaLa on H3K9ac levels in bovine early embryo; Fig. S4. Effects of GSKA and NaLa on H3K27ac levels in bovine early embryo; Fig. S5. Differential gene expression profile mediated by reduced histone lactylation levels in early bovine embryos. [file 40104_2026_1398_MOESM4_ESM.docx]

**Figure S1**


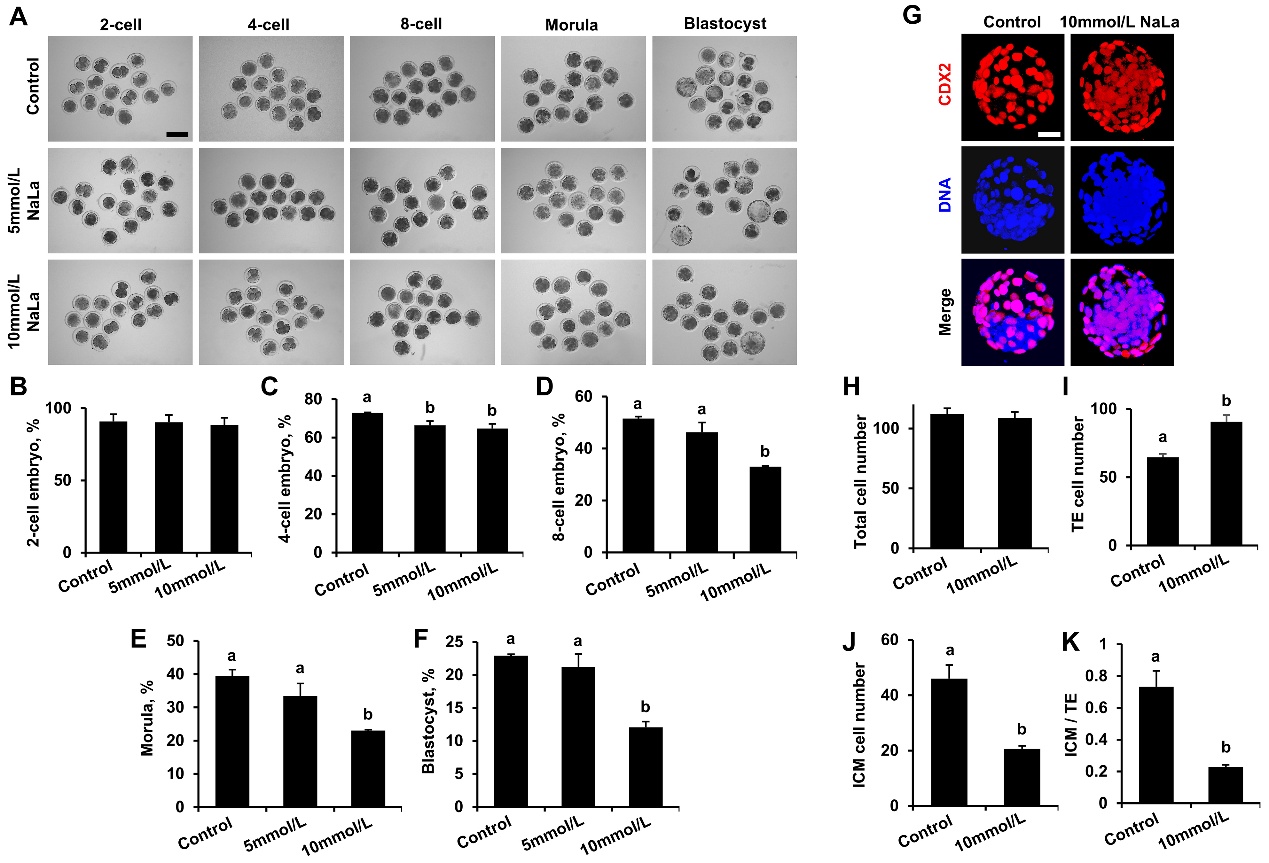


**Figure S1. Effects of NaLa on bovine early embryo development**

(**A**) Representative images of 2-cell embryo, 4-cell embryo, 8-cell embryo, morula, and blastocyst from the control, 5 mmol/L, and 10 mmol/L NaLa groups. Reference scale bar: 200 µm. Two-cell embryo, 4-cell embryo, 8-cell embryo, morula, and blastocyst were imaged at 30 h, 48 h, 60 h, 108 h, and 168 h post-IVF, respectively. The rates of 2-cell embryo (**B**), 4-cell embryo (**C**), 8-cell embryo (**D**), morula (**E**), and blastocyst (**F**) in the control (n = 143), 5 mmol/L (n = 144), and 10 mmol/L NaLa (n = 144) groups. *R* = 3. (**G**) Representative images of CDX2 immunofluorescence staining of blastocysts from the control and 10 mmol/L NaLa groups. Embryos were stained for CDX2 (red) and DNA (blue). Reference scale bar: 50 µm. The total cell number (**H**), TE cell number (**I**), ICM cell number (**J**), and ICM/TE cell ratio (**K**) for blastocyst in the control (n = 24) and 10 mmol/L NaLa (n = 24) groups. *R* = 3. Statistical comparisons of the data from Figures S1B–F were performed using one-way ANOVA with Tukey’s HSD post hoc multiple comparison test. Statistical comparisons of the data from Figures S1H–K were performed using Student's t test. Different letters on the bars indicate significant differences (*P* < 0.05).

**Figure S2**


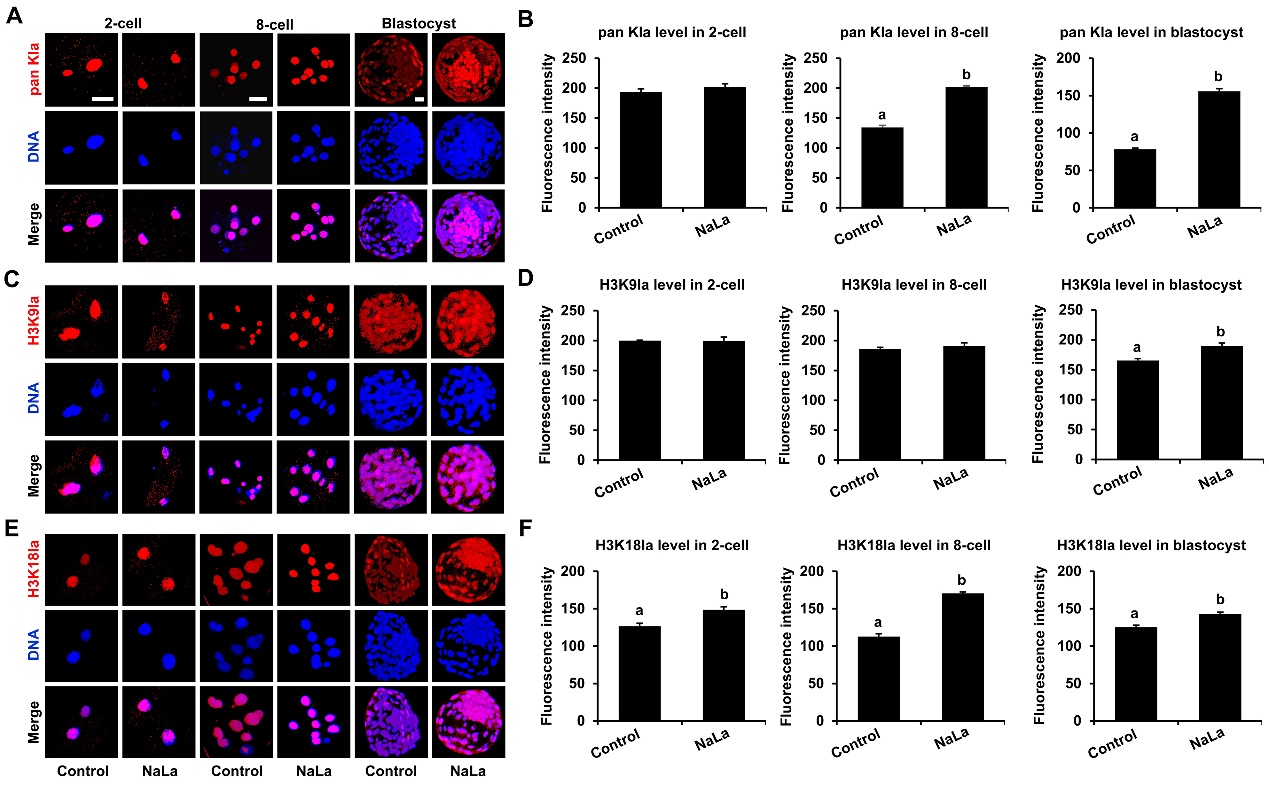


**Figure S2. Effects of NaLa on histone lactylation modification level in bovine embryo**

(**A**) Representative images of pan Kla immunofluorescence staining of 2-cell embryo, 8-cell embryo, and blastocyst from the control and 10 mmol/L NaLa groups. Embryos were stained for pan Kla (red) and DNA (blue). Reference scale bar: 25 µm. (**B**) The pan Kla fluorescence intensity level changes of 2-cell embryo in the control (n = 28) and 10 mmol/L NaLa (n = 25) groups. *R* = 3. (**C**) The pan Kla fluorescence intensity level changes of 8-cell in the control (n = 24) and 10 mmol/L NaLa (n = 24) groups. *R* = 3. (**D**) The pan Kla fluorescence intensity level changes of blastocyst in the control (n = 25) and 10 mmol/L NaLa treatment (n = 30) groups. *R* = 3. (**E**) Representative images of H3K9la immunofluorescence staining of 2-cell embryo, 8-cell embryo, and blastocyst from the control and 10mmol/L NaLa treatment groups. Embryos were stained for H3K9la (red) and DNA (blue). Reference scale bar: 25 µm. (**F**) The H3K9la fluorescence intensity level changes of 2-cell embryo in the control (n = 26) and 10 mmol/L NaLa (n = 24) groups. *R* = 3. (**G**) The H3K9la fluorescence intensity level changes of 8-cell embryo in the control (n = 25) and 10 mmol/L NaLa (n = 24) groups. *R* = 3. (**H**) The H3K9la fluorescence intensity level changes of blastocyst in the control (n = 26) and 10 mmol/L NaLa (n = 24) groups. *R* = 3. (**I**) Representative images of H3K18la immunofluorescence staining of 2-cell embryo, 8-cell embryo, and blastocyst from the control and 10 mmol/L NaLa treatment groups. Embryos were stained for H3K18la (red) and DNA (blue). Reference scale bar: 25 µm. (**J**) The H3K18la fluorescence intensity level changes of 2-cell embryo in the control (n = 28) and 10 mmol/L NaLa (n = 26) groups. *R* = 3. (**K**) The H3K18la fluorescence intensity level changes of 8-cell in the control (n = 25) and 10 mmol/L NaLa (n = 26) groups. *R* = 3. (**L**) The H3K18la fluorescence intensity level changes of blastocyst in the control (n = 24) and 10 mmol/L NaLa GSKA (n = 24) groups. *R* = 3. Two-cell embryo, 8-cell embryo, and blastocyst were collected at 30 h, 60 h, and 168 h post-IVF, respectively. Statistical comparisons of the data were performed using Student's t test. Different letters on the bars indicate significant differences (*P* < 0.05).

**Figure S3**


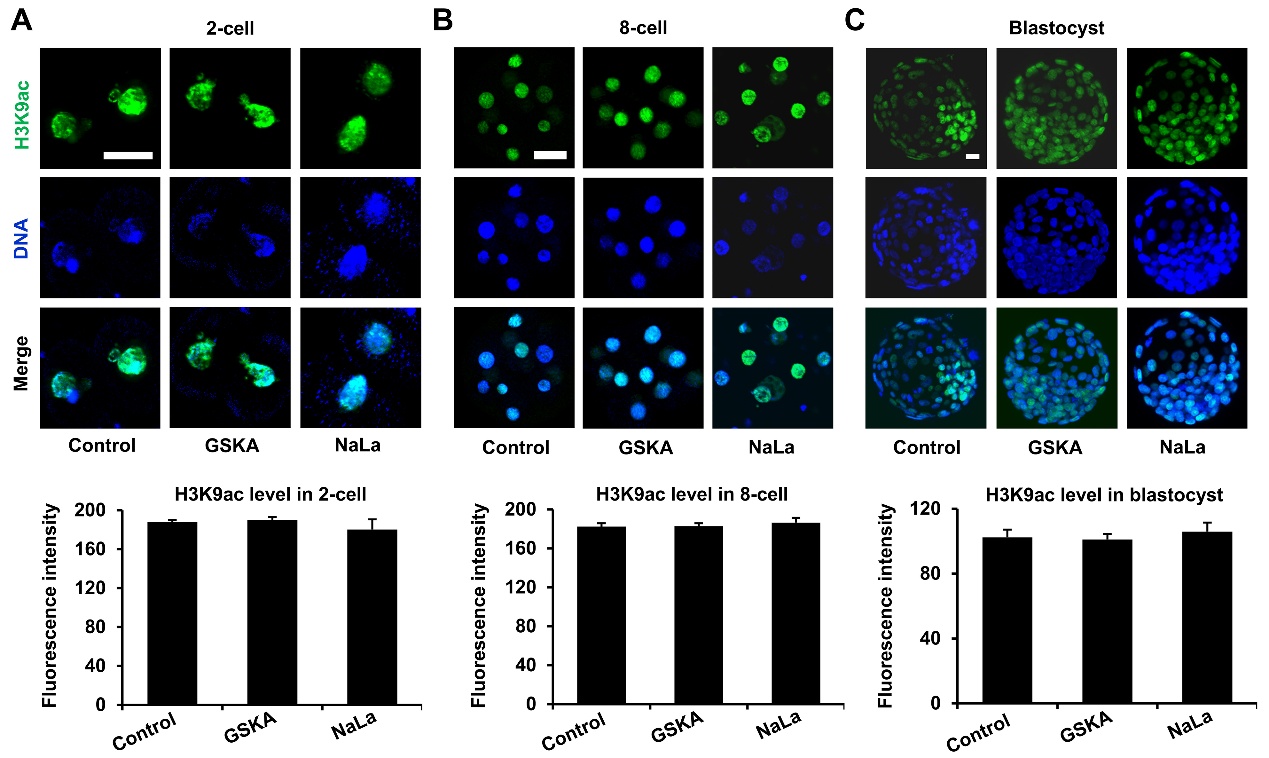


**Figure S3. Effects of GSKA and NaLa on H3K9ac levels in bovine early embryo**

(**A**) Representative images of H3K9ac immunofluorescence staining and fluorescence intensity level changes of 2-cell embryo in the control (n = 30), 200 pmol/L GSKA (n = 32), and 10 mmol/L NaLa (n = 34) groups. *R* = 3. Embryos were stained for H3K9ac (green) and DNA (blue). Reference scale bar: 25 µm. (**B**) Representative images of H3K9ac immunofluorescence staining and fluorescence intensity level changes of 8-cell embryo in the control (n = 25), 200 pmol/L GSKA (n = 28), and 10 mmol/L NaLa (n = 24) groups. *R* = 3. Embryos were stained for H3K9ac (green) and DNA (blue). Reference scale bar: 25 µm. (**C**) Representative images of H3K9ac immunofluorescence staining and fluorescence intensity level changes of blastocyst in the control (n = 28), 200 pmol/L GSKA (n = 26), and 10 mmol/L NaLa (n = 30) groups. *R* = 3. Embryos were stained for H3K9ac (green) and DNA (blue). Reference scale bar: 25 µm. Two-cell embryo, 8-cell embryo, and blastocyst were collected at 30 h, 60 h, and 168 h post-IVF, respectively. Statistical comparisons were performed using one-way ANOVA with Tukey’s HSD post hoc multiple comparison test. Different letters on the bars indicate significant differences (*P* < 0.05).

**Figure S4**


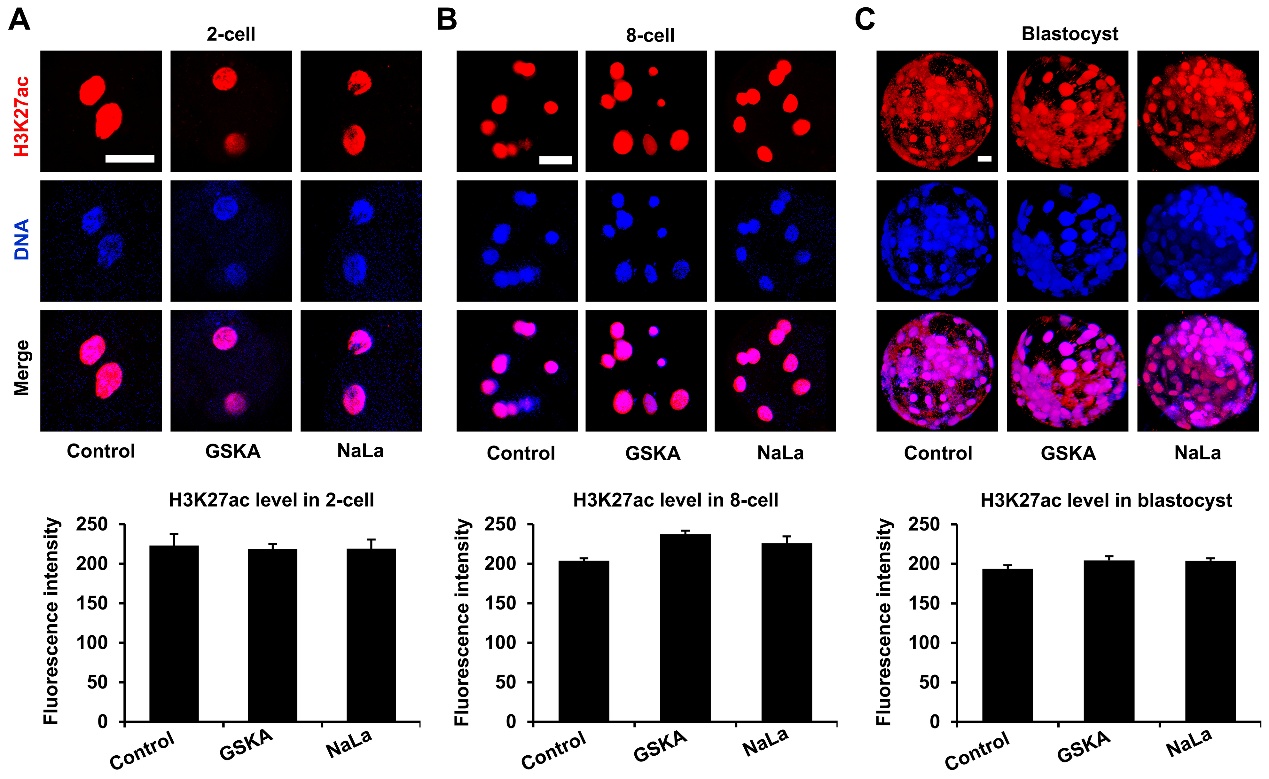


**Figure S4. Effects of GSKA and NaLa on H3K27ac levels in bovine early embryo**

(**A**) Representative images of H3K27ac immunofluorescence staining and fluorescence intensity level changes of 2-cell embryo in the control (n = 30), 200 pmol/L GSKA (n = 31), and 10 mmol/L NaLa (n = 25) groups. *R* = 3. Embryos were stained for H3K27ac (green) and DNA (blue). Reference scale bar: 25 µm. (**B**) Representative images of H3K27ac immunofluorescence staining and fluorescence intensity level changes of 8-cell embryo in the control (n = 28), 200 pmol/L GSKA (n = 25), and 10 mmol/L NaLa (n = 25) groups. *R* = 3. Embryos were stained for H3K27ac (green) and DNA (blue). Reference scale bar: 25 µm. (**C**) Representative images of H3K27ac immunofluorescence staining and fluorescence intensity level changes of blastocyst in the control (n = 26), 200 pmol/L GSKA (n = 24), and 10 mmol/L NaLa (n = 28) groups. *R* = 3. Embryos were stained for H3K27ac (green) and DNA (blue). Reference scale bar: 25 µm. Two-cell embryo, 8-cell embryo, and blastocyst were collected at 30 h, 60 h, and 168 h post-IVF, respectively. Statistical comparisons were performed using one-way ANOVA with Tukey’s HSD post hoc multiple comparison test. Different letters on the bars indicate significant differences (*P* < 0.05).

**Figure S5**


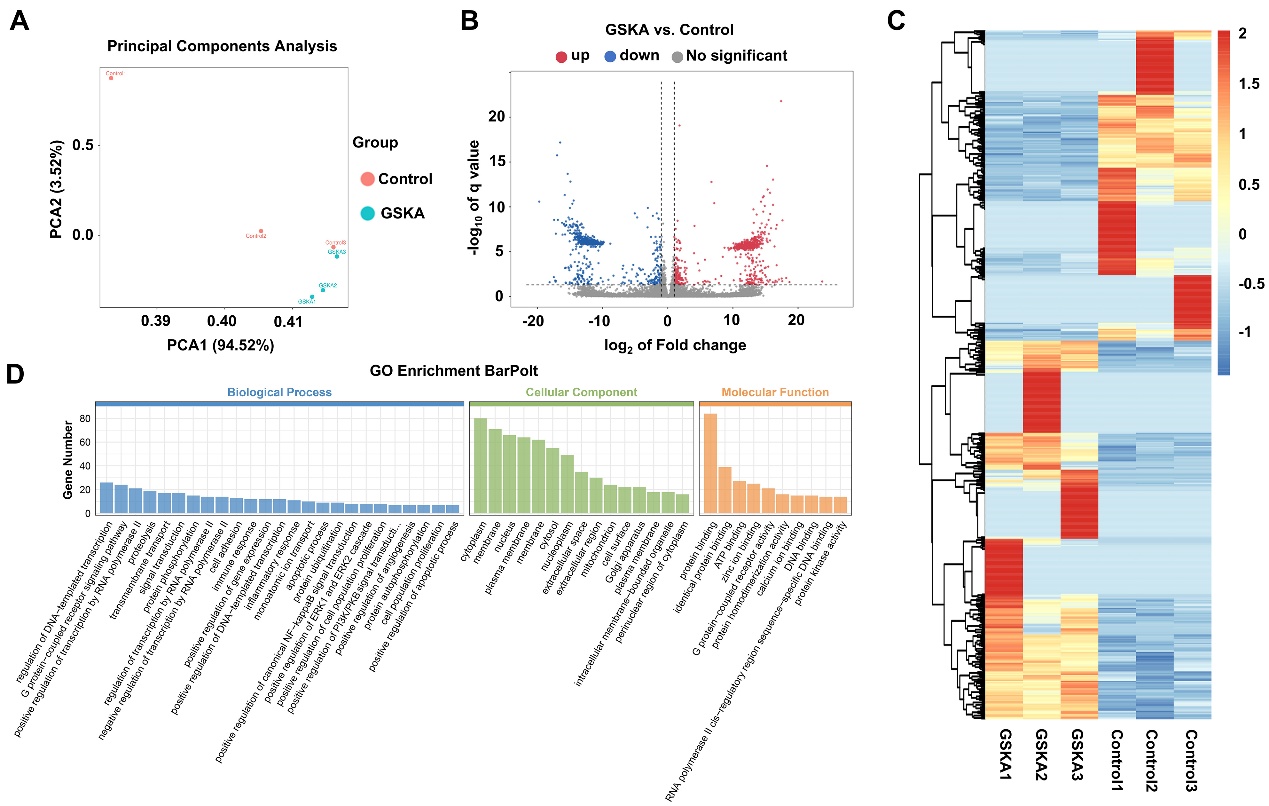


**Figure S5. Differential gene expression profile mediated by reduced histone lactylation levels in early bovine embryos**

(**A**) PCA cluster analysis of scRNA-seq data of 8-cell embryos in the control and 200 pmol/L GSKA groups. The light blue dots indicate the control group; the orange dots indicate the GSKA group. *R* = 3. (**B**) Volcano plot of genes in 8-cell embryos in the control and 200 pmol/L GSKA groups. The red dots represent significantly upregulated genes, and the blue dots represent significantly downregulated genes. *R* = 3. (**C**) Cluster analysis of genes in 8-cell embryos in the control and 200 pmol/L GSKA groups. Red indicates a relatively high expression level; blue indicates a relatively low expression level. The X-axis is log_2_(fold change). The Y-axis is log_10_(q value). *R* = 3. (**D**) GO enrichment bar plot of DEGs. The blue bars represent biological process terms; the light green bars represent cellular component terms; and the light orange bars represent molecular function terms. The X-axis is different terms. The Y-axis is the number of genes enriched in different terms.
